# Ceramic Matrix Composite Cyclic Ablation Behavior under Oxyacetylene Torch

**DOI:** 10.3390/ma17184565

**Published:** 2024-09-17

**Authors:** Hailang Ge, Xianqing Chen, Guangyu Li, Lu Zhang

**Affiliations:** 1 College of Mechanical and Electrical Engineering, Suqian University, Suqian 223800, China; 2 College of Energy and Power Engineering, Nanjing University of Aeronautics and Astronautics, Nanjing 210016, China

**Keywords:** SiC/SiC, high-temperature ablation, cyclic ablation, ablation behavior

## Abstract

To study the ablation properties and differences of plain-woven SiC/SiC composites under single and cyclic ablation. The ablation test of plain-woven SiC/SiC composites was conducted under an oxyacetylene torch. The results indicate that the mass ablation rate of cyclic ablation is lower than that of single ablation, whereas the line ablation rate is higher. Macro-microstructural characterization revealed the presence of white oxide formed by silica on the surface of the ablation center region. The fibers in the central region of the ablation were ablated layer by layer, and the broken fiber bundles exhibited a spiky morphology with numerous silica particles attached. The oxide layer on the surface and the silica particles on the fibers, which are in the molten state formed in the high-temperature ablation environment, contribute to resisting ablation. Thermal shock during cyclic ablation also played a role in the ablation process. The thermal shock causes cracks in the fiber bundles and matrix of the SiC/SiC composites. This study helps to apply SiC/SiC composite to complex thermal shock environments.

## 1. Introduction

SiC/SiC composites are characterized by their high specific modulus, high specific strength, and excellent high-temperature resistance [1,2,3]. They have received wide attention as components for the hot end of aero-engine [4,5,6]. Compared with high-temperature alloys, SiC/SiC composites have higher temperature resistance and can be operated at high temperatures for long periods of time [7,8,9]. Currently, they are widely used in turbines, combustion chambers of detonation engines, rocket engine nozzles, and components of heat exchangers [10,11,12]. Unfortunately, SiC/SiC composites generally suffer from ablation in high-temperature environments [13]. SiC/SiC ablation leads to pneumatic and heat transfer problems [14,15], which limits the application of these materials in high-temperature gas environments. Therefore, studying the ablation properties of SiC/SiC composites in service environments is important to solve these problems.

In recent years, many scholars have researched the ablation properties and mechanisms of C/C, C/SiC, and SiC/SiC composites in high-temperature environments [16,17,18,19,20]. It is generally recognized that ablation is caused by the combined effect of mechanical scouring, physical sublimation, and thermochemical reactions. The ablation behavior of the SiC matrix of C/SiC composites is mainly thermal decomposition and oxidation at lower than 2900 °C. The ablation behavior above 3550 °C is mainly sublimation [21]. SiO_2_ melt is generated on the surface of C/SiC materials during ablation, and it has three flow forms: droplet flow, liquid flow, and liquid film flow [22,23]. The same liquid layer flow exists on the surface of SiC/SiC composites [24]. The gas generated during ablation disrupts the continuity of the liquid film, which allows oxygen to erode the interior of the material and reduce its resistance to ablation. The thermal ablation process of C/SiC composites can be visually recorded with the help of a high-speed camera [25]. The camera reveals that the ablation of the material can result in the formation of a “pore” structure, surface cracks, and a “skeleton structure”. After ablation, the ablated area is divided into three zones according to the distribution of silica on the surface [26,27].

The above studies focus on the single ablation characteristics and mechanism of composites. In order to investigate the cyclic ablation characteristics of composites further, the ablation behavior of composites under different ablation environments is explored. The cyclic ablation of composites is mainly affected by the stresses caused by crystallization transformation during the heating–cooling process [28,29]. In cyclic ablation mode, the ablation characteristics of different materials are different. The mass loss of C/C-ZrC-SiC composites is lower than that of C/C-ZrC composites [30]. Considering the process of material heating, the higher the number of cyclic ablation, the lower the specimen surface temperature [31]. Due to the lower surface temperature of cyclic ablation, the cyclic ablation performance is better than that of single ablation. However, the repeated impacts of cyclic ablation will generate thermal stresses, which will lead to some cracks on the surface of the material [32]. Considering the effect of time on ablation, the ablation resistance of C/C-SiC composites is enhanced with time under single ablation and improves more significantly under cyclic ablation [33]. For the effect of adding different elements on the cyclic ablation performance, Zhang [34] found that composites containing 40 wt% aluminum have better ablation resistance during cyclic ablation. The cyclic ablation performance of C/SiC composites with added ZrC is superior to single ablation [35]. For the effect of airflow on the cyclic ablation process, Feng [36] found that a low-velocity airflow helps the diffusion and accumulation of ablation products, while a stronger airflow causes cracks and fragmentation on the surface of the composites.

Although SiC/SiC composites exhibit desirable ablation resistance, during cyclic ablation, the composites undergo rapid heating, cooling, and reheating. This can affect the stability of surface products exposed to thermal gradients and stresses generated by cyclic thermal loading. However, the ablation response of SiC/SiC composites subjected to cyclic ablation thermal shock under ultra-high-temperature ablation conditions has received less study. If the total ablation time is the same. The material has been ablated single and multiple times, so the ablation rate and the macro-micro morphology of the material surface should be the same. This paper focuses on the ablation behavior of plain-woven SiC/SiC composites under different ablation methods and compares the effect of single ablation and cyclic ablation on the ablation properties of composites. This study of cyclic ablation properties of SiC/SiC composites can complement studies on ablation properties of composites. It is valuable to apply ceramic matrix composites (CMCs) to the hot end parts of the aero-engine.

## 2. Materials and Methods

### 2.1. Material Parameters and Preparation

The Beijing Institute of Aeronautical Materials Research of China Aerospace Development (BIMR) supplied the materials used in this test. The test materials were prepared using chemical vapor deposition (CVD) and precursor impregnation pyrolysis (PIP). A plain weave was used to weave the silicon carbide fibers. Then, a flat surface topography was formed on the specimen surface by depositing SiC. The density of the material was 1.98 g/cm^3^. The sheets were machined into 30 mm × 30 mm × 3 mm specimens using waterjet cutting.

### 2.2. Ablation Test

**Figure 1:**
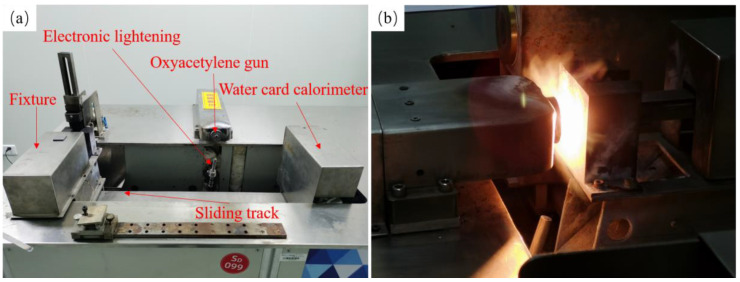
Ablation test rig (**a**) Ablation test rig composition, (**b**) Ablation test.

The national military standard protocol (GJB 323B-2018) [37] was used to carry out the oxy-acetylene ablation test. As shown in Figure 1a, the ablation test rig contains oxygen–acetylene gas, an oxyacetylene gun, a fixture, an electronic ignition system, a sliding track, and a water card calorimeter. A stationary bracket with water cooling is used to secure the specimen. The electronic ignition system enables the automatic ignition of the gun. A water card calorimeter (LWGY, Tianjin, China) is used to measure the heat flux of the gas. The PLC (Programmable Logic Controller) of the ablation system controls the gas flow and pressure. The flow rate and pressure of oxygen and acetylene are adjusted according to the target gas heat flux value, achieving automatic control of the oxygen and acetylene flow rate and pressure. The flow rates of oxygen and acetylene for the ablation test were 22,654 mL/min and 16,719 mL/min, respectively, and the pressures of oxygen and acetylene were 0.35 MPa and 0.08 MPa, respectively. The heat flux of the gas for the ablation test, measured by the water card calorimeter, was 4.25 MW/m^2^. Figure 1b shows the ablation test in progress. The distance between the oxyacetylene gun and the surface of the specimen was 10 mm, the nozzle diameter of the oxyacetylene gun was Φ2 mm, the flame width was approximately 4–5 mm, the maximum flame temperature was about 2900 °C [26], and the diameter of the circular area of the specimen on the stationary holder exposed to the gas environment was Φ25 mm. Cyclic and single ablation tests were conducted for plain-woven SiC/SiC composites. As shown in Table 1, the 1# specimen was ablated four times for 15 s, the 2# specimen was ablated two times for 30 s, and the 3# specimen was ablated once for 60 s. The weight of the specimen is measured using a precision electronic scale before the ablation test. It needs to be removed and re-measured for mass after each ablation. The specimen is then reinstalled for cyclic ablation.

After the ablation test was completed, the weight of the specimen was measured using a precision electronic scale with an accuracy of 0.1 mg. The thickness of the specimens before and after ablation was measured using an electronic thickness gauge with an accuracy of 1 µm. Due to the uneven temperature distribution of the oxyacetylene flame, there is a certain thermal gradient between the center of the flame and the edge of the specimen. The depth of the ablated surface of the specimen varies, so it is necessary to measure the thickness at three points in the ablated area of the specimen and then take the average value to calculate the line ablation rate of the ablated specimen. When performing cyclic ablation, the specimen weight and thickness need to be measured after each ablation. The mass ablation rate (MAR) and line ablation rate (LAR) are defined as follows [29]:

(1) $$MAR=\frac{m_{0}-m_{t}}{t}$$

(2) $$LAR=\frac{d_{0}-d_{t}}{t}$$

where $m_{0}$ and $m_{t}$ are the masses of the specimens before and after ablation, the $d_{0}$ and $d_{t}$ are the thickness of the specimen center region before and after ablation, and t is the ablation time.

### 2.3. Material Characterization

The microstructure of the specimens was observed by Scanning Electron Microscope (SEM). The scanning electron microscope used was FESEM, ZEISS Gemini SEM 300 (Carl Zeiss, Oberkochen, Germany). The specimens were analyzed using energy-dispersive X-ray spectroscopy (EDS).

## 3. Results and Discussion

### 3.1. Ablative Properties

**Figure 2:**
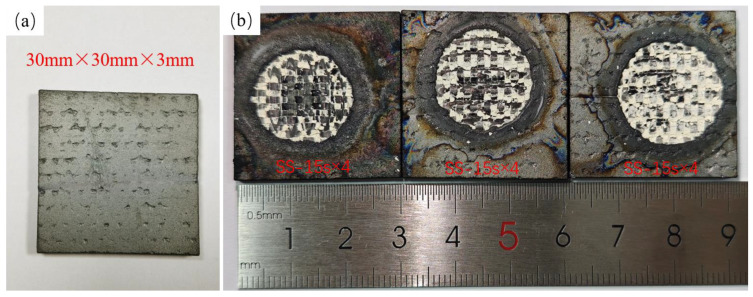
Surface morphology of specimens (**a**) Ablation specimen, (**b**) Surface morphology of ablated specimens.

The surface of the ablated specimen is shown in Figure 2a. A silicon carbide coating is deposited on the surface of the specimen. The coating has no large pits, and the surface is flat. The macroscopic morphology after single and cyclic ablation is shown in Figure 2b. The surface of the specimen shows significant recession, with obvious flow traces at the edges. The circular pit in the middle is covered with a white oxide layer, whose morphology is consistent with the form of a plain-woven structure. The white oxide layer of Specimen SS-15s×4 is detached the most, and the center region of this specimen experiences the most severe ablation damage. The white oxide morphology of Specimen SS-60s×1 is preserved the most complete, and it experiences less ablation damage. The main reason is that specimen SS-15s×4 was ablated four times, and during cyclic ablation, it proceeds through the process of cooling from high to low temperature, and the white silicon oxide on the surface will not be closely connected with the deep silicon carbide fiber due to thermal expansion and contraction. In the subsequent ablation process, the surface white oxide layer is eroded by the high-speed airflow of the spray gun before it has a chance to melt onto the surface of the specimen. This resulted in the internal silicon carbide fiber bundles being exposed to the high-temperature ablation environment, accelerating the ablation rate of the silicon carbide fibers in the central region. In contrast, the oxide layer generated during the single ablation test melted onto the surface and served to protect the internal silicon carbide fiber bundles.

**Figure 3:**
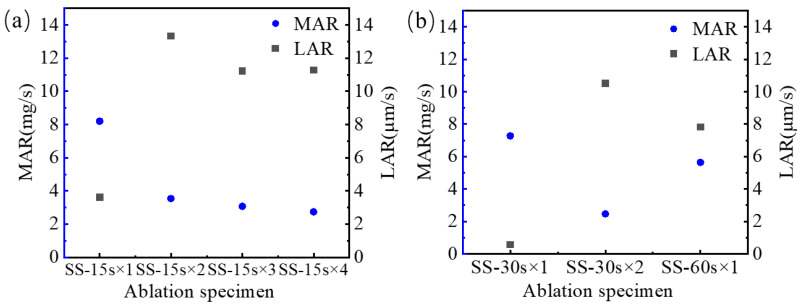
MAR and LAR of Cyclic and single ablation (**a**) Ablation rate of SS-15s×4, (**b**) Ablation rates of SS-30s×2 and SS-60s×1.

The mass ablation rate and line ablation rate of the single and cyclic ablations of SiC/SiC composites are listed in Table 2. The mass ablation rate and line ablation rate of single ablations are 5.63 mg/s and 7.82 µm/s, respectively. The mass ablation rate and line ablation rate in the cyclic ablation method exhibit a large difference between the initial and subsequent ablations. The initial mass ablation rates of specimens SS-15s×4 and SS-30s×2 were 8.19 mg/s and 7.27 mg/s, and the initial line ablation rates of each specimen were 3.6 µm/s and 0.57 µm/s. In the subsequent ablation process, the mass ablation rates of both specimens SS-15s×4 and SS-30s×2 decreased gradually while the line ablation rates increased. As can be seen from Figure 3, the mass ablation rate and line ablation rate for cyclic ablation varied greatly. The mass ablation rates at the first ablation of cyclic ablation are all higher, indicating that the mass loss in the initial period of ablation is greater, and the silicon carbide coating on the surface is directly sublimated or eroded. As the cyclic ablation continues, the mass ablation rate gradually decreases, which indicates that after the surface silicon carbide coating is stripped, the inner silicon carbide fiber is not easy to strip, and the silicon carbide fiber has good ablation resistance characteristics in terms of mass ablation rate. In contrast to the mass ablation rate characteristics, the linear ablation rate at the first ablation of the cyclic ablation is lower, indicating that the material recedes less at the beginning of the ablation. As the cyclic ablation continues, the linear ablation rate gradually increases. The reason is that after the surface silicon carbide coating is gradually stripped, the silicon carbide fibers in the central region of ablation are severely damaged, and the silicon carbide fibers are less resistant to ablation in terms of the linear ablation rate. The mass ablation rate and line ablation rate of single ablation are relatively stable. The SiC/SiC composites withstood long-term thermal loads and torch flushing without repeated thermal shocks and showed desirable ablation resistance [38].

### 3.2. Ablation Behavior

**Figure 4:**
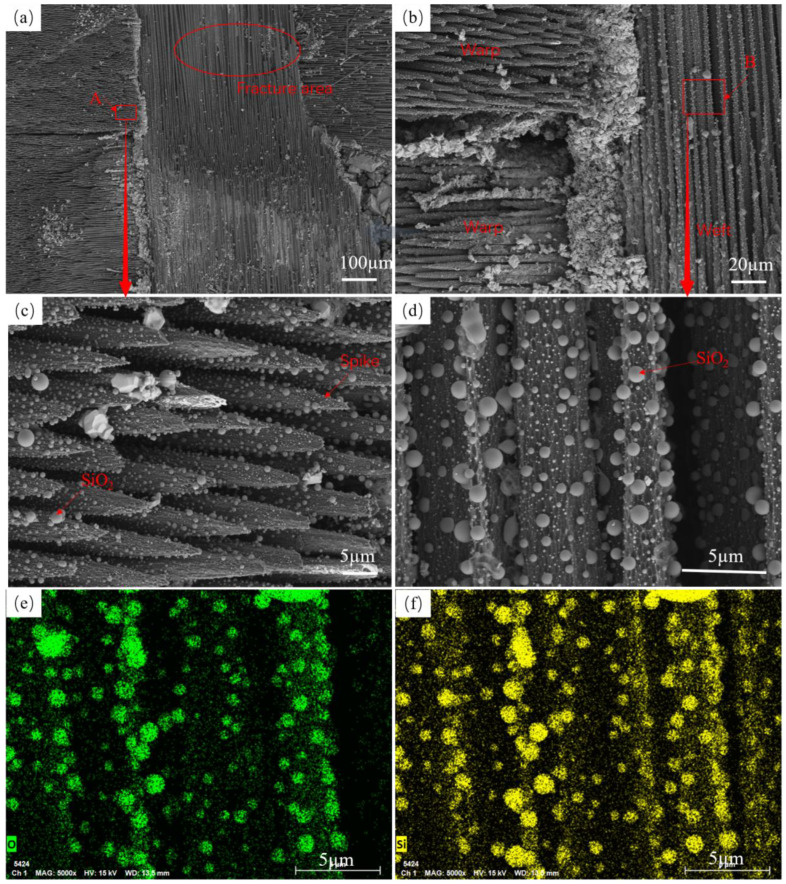
Morphology of the center area of SS-60s×1 ablation (**a**,**b**) Structure of SS-60s×1 after ablation, (**c**) Enlarged view of fiber breaks, (**d**) Enlarged view of fiber bundles, (**e**,**f**) EDS maps of fiber bundles.

The overall morphology of fiber bundles in the central area of the ablation of specimen SS-60s×1 and the microscopic morphology of fibers are shown in Figure 4. At the junction of warp and weft yarns in the plain-woven structure, the fiber bundles were gradually ablated. The characteristics similar to the multilayer distribution of the plain-woven structure are shown in Figure 4a,b. The red oval area indicates the fiber bundle of the weft yarn of the plain-woven composite. The fiber bundles are first fractured and damaged during the ablation process, and the ablation recedes continuously along the direction of the fiber bundles, eventually leading to the complete ablation of the outermost region in direct contact with the flame. Ablation damage is characterized by layer-by-layer ablation from the outside to the inside. As shown in Figure 4a, the warp yarns above the junction of warp and weft yarns have been completely ablated. There are still ablated residual warp yarns on the left side of this junction position, and the weft yarns at the junction are becoming fewer and will be completely ablated in the subsequent ablation process, continuing to ablate the next layer of warp yarns. The A region in Figure 4a is enlarged, as shown in Figure 4c. The warp fiber is broken after ablation, and the broken fiber gradually forms a spike-like shape under the continuous ablation of the flame. There are a large number of spherical silica particles on the surface of the spike-like fibers, which is due to the low wettability of liquid silica on the silicon carbide fibers [39,40,41]. The liquid material melts and becomes larger in a high-temperature environment, and during cooling, it shrinks to a spherical crown. This irregular spherical structure continues to change with the addition of heat and eventually evolves into a spherical structure driven by surface tension. The generation of spherical particles mainly undergoes nucleation and collisional growth processes [23], where loose silica shrinks into clusters unevenly distributed at the ends of ablation fractured fibers and on the fiber surface. Figure 4d is an enlarged view of the B area in Figure 4b, showing that the weft fibers are not broken after ablation. It can be seen that there is a fiber in the middle has a complete shape, a fiber on the left side is about to be completely ablated, and the fiber on the right side has a diameter of only 2 µm, indicating that the fiber in the surface layer will gradually become thinner under the high-temperature ablation environment. A large number of white dots and spherical particles are attached to the fiber surface, and the EDS scans of this region, shown in Figure 4e,f, indicate that the white dots and spherical particles are silica.

**Figure 5:**
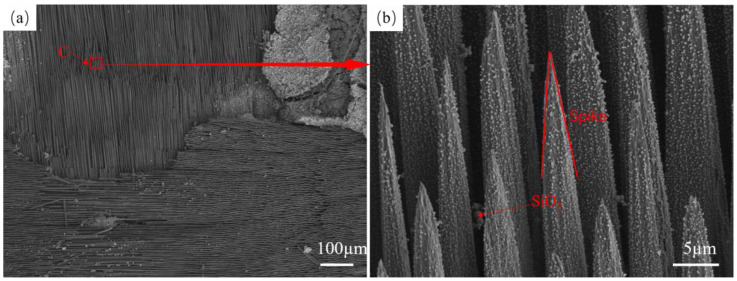
SS-30s×2 microscopic morphology (**a**) Macroscopic damage structure of SS-30s×2 after ablation, (**b**) Spike morphology of fiber fracture.

**Figure 6:**
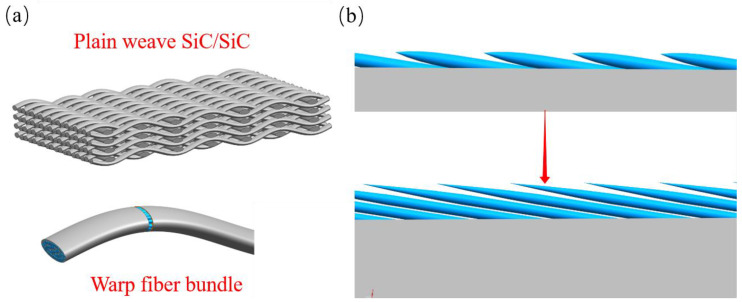
Fiber spike morphology and formation process in the central area of ablation (**a**) Plain-woven SiC/SiC composites, (**b**) Formation process of fiber fracture spike shape.

As shown in Figure 5a, the fiber bundles in the laminar shape in the central region of the SS-30s×2 ablation of the specimen were gradually damaged and fractured. After enlarging the C area in Figure 5a, as shown in Figure 5b, the morphology of the fiber bundle after fracture can be clearly seen.The ablation morphology in the center region of the fiber fracture exhibits a typical spike-like morphology, with fine silica particles uniformly distributed in the fiber spikes. Du [26] observed a similar needle-like morphology in the ablation experiments of 2.5D SiC/SiC composites. The reason is that different ablation reactivities of the matrix and fibers result in a weaker phase reacting first, which leads to differences in microstructural recession. The spike-like morphology can be specifically derived from the model proposed by Lachaud [42] for the relationship between the radius R of the micro-structured fibers and the height h of the post-ablation plane. Assuming that the fibers are perpendicular to the incoming direction, the fibers, matrix, and interface recede steadily, and the fibers are prone to forming needles when the matrix and interface have a higher recession velocity compared with the fibers. From Figure 5b, it can be seen that the substrate in the surface layer is completely ablated, and the receding velocity of the substrate is significantly higher than that of the fiber, which is the main reason for the formation of fibers in the shape of spikes. The process of spike-like fiber formation is shown in Figure 6. First, cracks are generated at the junction of warp and weft yarns in the 2D braided structure. The angle between the weft yarn and the oxyacetylene torch is 90°, and the angle between the warp fiber bundle and the oxyacetylene torch is close to 90°. When the warp fiber bundle gradually breaks, the matrix portion of the surface is consumed first, and then the fiber bundle fracture is gradually ablated. The morphology of the fiber fracture during this period is shown by the short fiber spikes in Figure 6b, and the ablated matrix recedes further as the ablation continues. As shown by the red arrow in Figure 6b, The fibers that lost the protection of the silicon carbide matrix gradually formed finer and sharper spikes during the ablation process.

### 3.3. Comparison of Cyclic and Single Ablation

**Figure 7:**
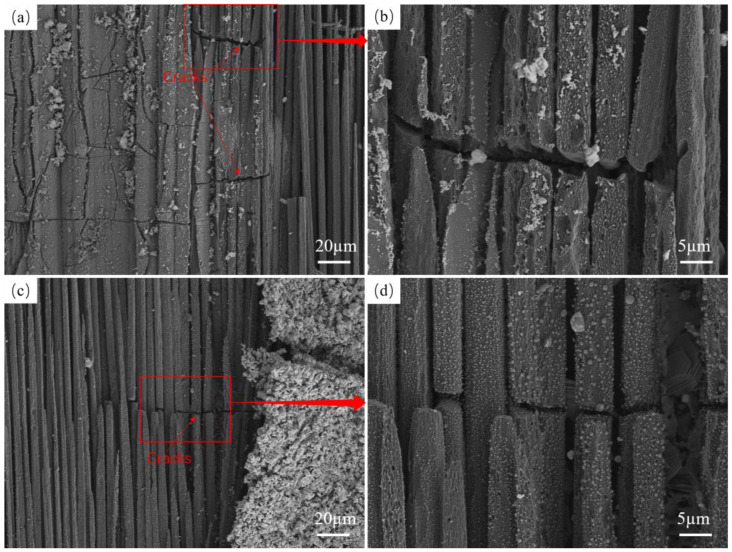
Cyclic ablation crack morphology (**a**,**b**) SS-15s×4 crack morphology, (**c**,**d**) SS-30s×2 crack morphology.

The crack distribution of specimen SS-15s×4 is shown in Figure 7a. There is a layer of matrix on the surface, and there are a large number of cracks on the matrix. The magnified view of the area with a larger crack width, as shown in Figure 7b, is about 50 µm in length and width about 3 µm in width. The crack distribution of specimen SS-30s×2 is shown in Figure 7c. There is only one crack with a length of about 50 µm and a width of about 1 µm. The number of cracks and the size of cracks in specimen SS-15s×4 are obviously larger than those in specimen SS-30s×2. By analyzing the micro-morphology, specimen SS-60s×1 does not have similar penetration cracks. This indicates that the specimens after cyclic ablation will produce cracks, and the number and size of cracks increase with an increase in the number of cyclic ablations. This is mainly due to the fact that repeated thermal shocks during cyclic ablation lead to thermal stresses. In addition, the number of cracks in the cyclic ablation specimens is less, and the size is smaller. This occurs mainly because the fiber and matrix of SiC/SiC composites are silicon carbide materials. The coefficient of thermal expansion of the silicon carbide fiber and the substrate are the same [43]. So, compared with composites where the fiber and matrix are not the same material, SiC/SiC composites can effectively resist the effect of thermal shock and have a better potential for application in a cyclic ablation environment.

**Figure 8:**
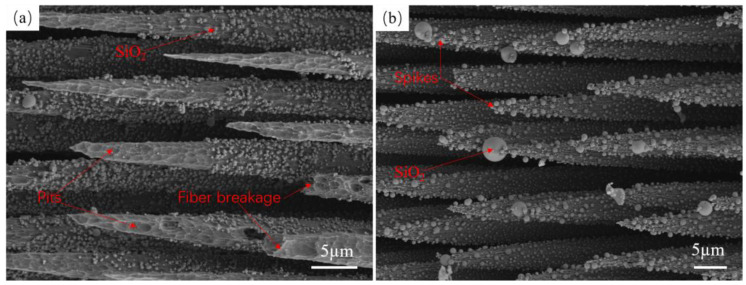
Cyclic ablation and single ablation spike morphology (**a**) SS-15s×4 spike morphology, (**b**) SS-60s×1 spike morphology.

Figure 8a,b show the spike-like fiber morphology of the ablation specimens SS-15s×4 and SS-60s×1, respectively. Irregular pits of different sizes appeared on the surface of the fiber spikes of the cyclic ablation specimens, and the larger pits could easily lead to fiber fracture. The internal surface of the pits was smooth, and there was no granular silica adhering in or around the pits. Fine silica particles were present at locations other than the fiber spike area. The single-ablation spikes and fiber surfaces have very small pits, the size of which is significantly smaller than that of the pits on the cyclically ablated spikes. The fiber surfaces have uniformly distributed silica particles and large silica particles form at the end of the spikes. In addition, the size of the silica particles is significantly larger than that of the cyclically ablated fiber surface particles. The ablation process mainly involves sublimation and mechanical stripping. In the single ablation environment, the molten silica forms a silica film to protect the fiber, which is uniformly distributed on the fiber surface and even at the fiber breaks. Under continuous ablation, small silica particles are continuously fused to form large particles. However, in the cyclic ablation environment, the initial ablation time is shorter, and the temperature is lower. The matrix oxidation in the central region of ablation generates silica, which does not have sufficient time or higher temperature to melt and increase in size, so large silica particles can not form on the fiber surface. In a high-temperature and erosive environment, the molten state of silica does not have time to distribute in the fiber spikes. At high temperatures, a stable silica film cannot form, and the fiber fractures are constantly eroded. Fine silica particles are eroded first, leading to the formation of pits of different sizes on the surface of the spikes.

## 4. Discussion

The ablation of CMCs under an oxyacetylene torch is a complex process involving oxidation, evaporation, mechanical erosion, and thermal decomposition [21]. During the ablation process, the surface of the specimen is directly exposed to an oxidizing environment at high temperatures, and the silicon carbide deposited on the surface of the SiC/SiC composites rapidly oxidizes to produce a protective silica “coating.” The temperature of the ablation center is about 2900 °C, so a small portion of the silica vaporizes at high temperatures, causing the surface temperature to drop. Most of the silica accumulates to protect the nearby silicon carbide matrix and fibers from oxidation or spalling and to prevent oxygen penetration; thus, the ablation resistance of the composites is enhanced over time. The mass and line ablation rates of the SiC/SiC composites by single ablation show excellent ablation resistance, and no microcracks were observed on the surface of the ablated specimens. Unlike single ablation, there is a destructive factor: thermal shock is involved in the cyclic ablation process. Oxyacetylene ablation tests used by Zhao [31] and Xie [32] found that the thermal stresses generated by the cyclic ablation process caused damage to the material. These findings are in agreement with the present test results, i.e., under the repeated impact of the oxyacetylene torch, significant thermal stresses were generated between the ablation products and the substrate. A certain number of cracks were present on the surface of the cooled specimens, and the bond between the ablation products and the substrate was not tight. The silica produced during the ablation process is affected by aerodynamic forces, thermal stresses, and other factors, and the adhesion is reduced during the cyclic heating process, which makes it easier to be eroded, accelerating the ablation rate in the region directly opposite to the oxygen alkyne torch; and resulting in a high rate of cyclic ablation in the line of ablation. The whole non-ablation center region was not stripped by thermal shock due to the smaller thermal shock, and the ablation products were well bonded to the substrate. In addition, the time of the composite material subjected to high temperatures in the cyclic ablation process is shorter than that of single ablation, resulting in a lower rate of mass loss in the non-ablative center region, which leads to the overall rate of mass loss in the cyclic ablation lower, so the mass ablation rate of cyclic ablation is lower than that of single ablation.

The number of cycles carried out in this experiment was two and four times, respectively. The degree of damage to the specimens after cyclic ablation is significantly larger than that of single ablation. SiC/SiC composites applied to the engine hot end structure are actually subjected to a greater number of thermal cycling impacts, and the damage to the composite structure may be more serious. The application of SiC/SiC to service environments with a higher number of thermal cycles requires further research in the future.

## 5. Conclusions

In this study, cyclic ablation of SiC/SiC composites at 15 s × 4, 30 s × 2, and single ablation tests at 60 s × 1 were completed. The cyclic ablation behavior of SiC/SiC composites under ultra-high-temperature conditions is revealed. The main conclusions are as follows:

(1) The mass ablation rate and line ablation rate of single ablation of plain-woven SiC/SiC composites are 5.63 mg/s and 7.82 µm/s, respectively. The initial mass ablation rate of the specimens SS-15s×4 and SS-30s×2 is 8.19 mg/s and 7.27 mg/s, and the initial line ablation rate is 3.6 µm/s and 0.57 µm/s, respectively. The average mass ablation rate of cyclic ablation is lower than that of single ablation, and the average line ablation rate is higher than that of single ablation.
(2) Macro-microstructural characterization shows that the central region of the laminar structure of the ablated fiber bundles gradually shows ablation fracture, and the fibers are spiky after fracture. Cyclic ablation results in smaller silica particles on the fiber surface than those observed in single ablation.
(3) There is an additional destructive factor in the cyclic ablation process. Thermal shock is also involved in the ablation process. The ablation product and the substrate experience thermal stresses that can produce cracks. Aerodynamic forces and thermal stresses during the ablation process reduce the adherence of the silicon dioxide. This accelerates the ablation of the region facing the oxyacetylene torch.

## Figures and Tables

**Table 1:** Ablation specimen numbers and ablation tests.

Specimen	Ablation Method	Number	Ablation Time/s	Cyclic Times
1#	Cyclic	SS-15s×1	15	1
		SS-15s×2	15	2
		SS-15s×3	15	3
		SS-15s×4	15	4
2#	Cyclic	SS-30s×1	30	1
		SS-30s×2	30	2
3#	Single	SS-60s×1	60	1

**Table 2:** SiC/SiC ablation rate results.

Specimen	Ablation Time/s	Mass before Ablation/mg	Mass after Ablation/mg	MAR ^1^/(mg·s^−1^)	Thickness before Ablation/µm	Thickness after Ablation/µm	LAR ^2^/(µm·s^−1^)
SS-15s×4	0–15	6314.8	6192	8.19	2967	2913	3.6
	15–30	6192	6139	3.53	2913	2713	13.33
	30–45	6139	6093	3.07	2713	2545	11.2
	45–60	6093	6052	2.73	2545	2376	11.27
SS-30s×2	0–30	6454.1	6236	7.27	2975	2958	0.57
	30–60	6236	6162	2.47	2958	2643	10.5
SS-60s×1	0–60	6406	6068	5.63	2964	2495	7.82

^1^ Mass ablation rate; ^2^ Line ablation rate.

## Data Availability

The original contributions presented in the study are included in the article, further inquiries can be directed to the corresponding author.
